# Back Numbers Wanted

**Published:** 1904-05

**Authors:** R. H. Harrison

**Affiliations:** Chairman; Columbus, Texas


### Back Numbers Wanted.

COLUMBUS, TEXAS, May 9, 1904.

*F. E. Daniel, M. D., Publisher Texas Medical Journal, Austin, Texas.*

MY DEAR DOCTOR: At the recent session of the Texas State Medical Association there was appointed a committee to collect and republish a number of volumes of its earlier transactions, that are about out of print, or so mutilated as to be unavailable. The volumes that the committee is desirous to secure are volume 1, published in 1871 or 1872, and, second, the transactions of 1872, 1873, 1874 and 1875, published in one volume; third, transactions for 1876, 1877, 1878, 1879 and 1880. Any member of the profession who can supply any one or more of these transactions, even if they are not in perfect order, will confer a favor upon the committee.

R. H. HARRISON, M. D.,
Chairman.

